# Loot

**Published:** 1870-12

**Authors:** 


### Loot.

***Value of Public Urinals.***

The sanitary committee of the Rochdale corporation have recently erected a public urinal, so constructed as to collect the product, which is extensively used for scouring cotton cloth. The cost was £161, and £8 8s. has been received as rental for the year. This, however, is greatly below the value, the ascertained product being upwards of 20,000 gallons a year. The committee anticipate, in future, a good income from these constructions.—*Lancet.*

Born on the 4th day of July, A. D. 1870, 5 minutes before 12 o'clock, M., in the village of Colon, St. Joseph Co., Mich., a female child, with the left fore-arm amputated, the stump healed by cicatrix; daughter of Margaret Bradley, wife of Nelson N. Bradley. Nathan Mitchell, M.D., Accoucheur.

***Vaccination in the Italian Army.***

Dr. Baroffio, in his report on vaccination in the Italian army in 1868, reports that the number vaccinated was about 57,000. The good effects of vaccination and revaccination are constantly becoming more apparent. The mortality from small pox in the Sardinian army, which formerly was nearly 7 per cent., in 1863 had fallen to 4 per cent., and in 1868 was little more than 3½. Dr. Baroffio expresses a favorable opinion of the plan of taking the lymph from healthy and strong infants, and then transmitting it from arm to arm among the soldiers.—*Brit. Med. Jour.*

***Application for Cancer.***

An Italian medical journal recommends the following application for cancer of the breast: Concentrated acetic acid, 15 parts; creasote, 3¾ parts; water 450 parts. A case is mentioned, in which a cancer was removed and cicatrization completed in six weeks. The application was made on lint, four or five times daily. —*Dental Times.*

Dr. S. H. Bennett recommends the following in the nausea and vomiting of phthisis:

| | | |
|---|---|---|
| ℞ | Naphthæ Medicinalis, - - - - - | f. ʒ i; |
| | Tinct. Cardamoni Comp., - - - - - | f. ℥; |
| | Mist. Camphoræ, - - - - - - | f. ℥ vii. |

M. Ft. Mist.

Sig.—A tablespoonful every four hours.

***Small Pox in Paris.***

The number of deaths in Paris from small pox is daily increasing. From the 17th to the 23rd April there were 132 deaths, and 166 from the 24th to the 30th. These results are surprising, in view of the great number of revaccinations which have taken place within four or five months past. The documents presented to the Academy by M. Vernois would lead one to impute the numerous cases of unsuccessful revaccination to vaccine taken from the animal, and to find, in this fact, the principal reason for the growing increase of the actual epidemic. It is, therefore, more urgent than ever to return to the vaccine of Jenner.—*Lyons Medical.*

***Temperature in Scarlatina.***

Dr. E. L. Fox, in some admirable clinical observations on the temperature of disease, says: "We must give a guarded prognosis in cases of scarlatina with very high temperature, but unfavorable cases are not always marked by extreme elevation of the mercury. Death will be ushered in by a high temperature, if it occurs during the eruptive period of the disease, but even then the thermometer may fall just before the fatal event." He thinks that a high temperature will be found to precede death from complications, such as scarlatinal rheumatism, pneumonia, arachnitis, and nephritis, while a fatal termination dependent on septiæmia may be accompanied generally, but not always, by a lower temperature. The pulse bears little definite relation to the temperature in scarlatina. A rise of temperature often occurs during desquamation, but this is not always the case. Acute tubal nephritis, of which albuminuria and hæmaturia are the evidences, will cause a rise of temperature, even to the maximum point of the original disease.—*Medical Times and Gazette.*

***Chinese Medication.***

Ling Wau, a Celestial charlatan, has taken up his abode in New York, and advertises not only an imported materia medica of three hundred "remarkable Chinese medicines," but imported apothecaries to dispense them. In this connection it may be of interest to remark, that San Francisco custom-house authorities recently seized an invoice of "remarkable Chinese remedies," consisting of pickled monkeys, dried toads, and such like medicinal articles. An elegant Mongolian prescription might read:

℞ Pulv. unguis pollicis pedis, - - - - - grs. v;
Bufonis exsiccati rasi, - - - - - - ʒ j;
Caudæ soricis ustæ, q. s. ad gratum saporem.

Tritura bene, et in chartulis v. divide, quarum capiat æger unam bis in die.

—*Medical Gazette.*

### *The Slain in War.*

What to do with the slain in the war, has occupied the attention of the *Gazette Hebdomadaire*, and we are glad to see that some of the writers are prepared to advocate burning as superior to burying. The ancient plan had many advantages from a sanitary point of view, and we should welcome any discussion which might tend to remove the objections of modern Europe to any mode of disposal of the dead that might be better for the living than that now practiced.—*Med. Press and Circular.*

### *Hair Dye.*

Mr. McDonald communicates to the *Am. Jour. of Pharmacy*, this, as a "Brown Hair Dye:"

"To those whose consciences will permit them to recommend such preparations to their customers, I submit the following formula—

| | | |
|---|---|---|
| ℞ | Acetate of Lead, - - - - - | ʒ ij; |
| | Hyposulphite of Soda, - - - - | ʒ j; |
| | Rose (or other perfumed) Water, - - - | ℥ xiv; |
| | Glycerin, - - - - - - | f ℥ ij; |

Dissolve the acetate of lead and hyposulphite of soda in separate portions of the perfumed water, filter separately, mix the solutions, and add the glycerin.

"In a short time after it is made, the solution will become slightly turbid. This may arise, either from a small quantity of air which is contained in the liquid, or, perhaps, from the presence of a small amount of oxalic acid with which glycerin is said to be sometimes contaminated. It, however, soon becomes clear by deposition of a minute quantity of dark powder (sulphuret of lead), and remains so, so long as the bottle is kept tightly corked. I think it is quite as harmless as the ordinary lead and sulphur dyes, but this is, at the best, only a left-handed recommendation."

### *Tincture of Erigeron in Alveolar Hæmorrhage.*

Mr. Perkins states, (*Dental Cosmos*, Aug. 1870,) that the tincture of erigeron is more efficacious than Monsel's salt for arresting the troublesome hæmorrhage sometimes following extraction of teeth.

### *Small Pox in Russia.*

In Russia, vaccination is not compulsory. According to official returns, 10,350,000 persons have died of small pox in that country during the last seventy years. What say our friends of the Anti-vaccination League to this?—*Lancet.*

